# Developmental Programming of Capuchin Monkey Adrenal Dysfunction by Gestational Chronodisruption

**DOI:** 10.1155/2018/9183053

**Published:** 2018-08-13

**Authors:** Hans G. Richter, Natalia Mendez, Lorena Abarzua-Catalan, Guillermo J. Valenzuela, Maria Seron-Ferre, Claudia Torres-Farfan

**Affiliations:** ^1^Instituto de Anatomía, Histología y Patología, Facultad de Medicina, Universidad Austral de Chile, Valdivia, Chile; ^2^Laboratorio de Obstetricia y Ginecología, Centro de Investigaciones Médicas, Pontificia Universidad Católica de Chile, Santiago, Chile; ^3^Department of Women's Health, Arrowhead Regional Medical Center, Colton, CA, USA; ^4^Programa de Fisiopatología, Instituto de Ciencias Biomédicas (ICBM), Facultad de Medicina, Universidad de Chile, Santiago, Chile; ^5^Centro Interdisciplinario de Estudios del Sistema Nervioso, Universidad Asutral de Chile, Valivia, Chile

## Abstract

In the capuchin monkey (*Cebus apella*), a new-world nonhuman primate, maternal exposure to constant light during last third of gestation induces precocious maturation of the fetal adrenal and increased plasma cortisol in the newborn. Here, we further explored the effects of this challenge on the developmental programming of adrenal function in newborn and infant capuchin monkeys. We measured (i) plasma dehydroepiandrosterone sulphate (DHAS) and cortisol response to ACTH in infants with suppressed endogenous ACTH, (ii) plasma DHAS and cortisol response to ACTH* in vitro*, and (iii) adrenal weight and expression level of key factors in steroid synthesis (StAR and 3*β*-HSD). In one-month-old infants from mothers subjected to constant light, plasma levels of cortisol and cortisol response to ACTH were twofold higher, whereas plasma levels of DHAS and DHAS response to ACTH were markedly reduced, compared to control conditions. At 10 months of age, DHAS levels were still lower but closer to control animals, whereas cortisol response to ACTH was similar in both experimental groups. A compensatory response was detected at the adrenal level, consisting of a 30% increase in adrenal weight and about 50% reduction of both StAR and 3*β*-HSD mRNA and protein expression and the magnitude of DHAS and cortisol response to ACTH* in vitro*. Hence, at birth and at 10 months of age, there were differential effects in DHAS, cortisol production, and their response to ACTH. However, by 10 months of age, these subsided, leading to a normal cortisol response to ACTH. These compensatory mechanisms may help to overcome the adrenal alterations induced during pregnancy to restore normal cortisol concentrations in the growing infant.

## 1. Introduction

In a complicated pregnancy, adverse conditions are imposed on the developing fetus. In order to cope, the fetus modifies its developmental program to allow for adaptation to the ensuing new physiological contexts as a newborn, infant, and adult. Mounting experimental and epidemiological data has shown that detrimental conditions experienced* in utero* have negative effects that become apparent in adult life [1–5].

As most species, human beings are subjected to environmental stressors, which continuously shape their evolutionary trajectory. Industrialized society is engaged in a myriad of activities that generate environmental pollution including particulate matter, high-level noise, and distorted lighting schedules (such as artificial light at night, shift-work, and jet lag). There is little doubt that the lack of adequate adaptive responses to these modern-life stressors is likely linked to chronic disease [6–9]. However, the effects of manipulating the most reliable signal of our life, the photoperiod [10], as well as its role in the adrenal programming, has not been extensively studied. The adrenal gland is a key organ fully involved in homeostasis, contributing to sustain an adequate balance of several physiological functions, even at dissimilar contexts across life [8, 11, 12]. Thus, early programming or anomalous setting of the adrenal gland may play an important role in development of diseases prevalent in our modern society. Although the connection between exposure to an altered photoperiod, pregnancy, and fetal outcome is not fully understood, there is mounting evidence linking preterm delivery and low birth weight in pregnancies subjected to shift-work [13–16]. Alterations of maternal physiology by shift-work, as suggested by an increased incidence of metabolic syndrome, may affect the fetus [16]. Similarly, we and others have recently shown that maternal chronodisruption in rats is translated to the offspring, inducing widespread negative consequences in the adult offspring's health including heart hypertrophy, decreased glucose tolerance, decreased spatial memory, and increased blood pressure and heart rate variability [17–21]. Additionally, animals gestated in chronodisruptive conditions, but raised in a normal photoperiod immediately after birth, have been shown to alter glucocorticoid circadian rhythms and a lack of nocturnal increases in melatonin [21].

In primates, maternal exposure to constant light (as a model of gestational chronodisruption) during the last third of gestation induced precocious maturation of the fetal primate adrenal [22] and resulted in increased plasma cortisol concentrations in the nonhuman primate soon after birth [23]. Interestingly, primate newborns gestated in constant light lack a synchronized circadian rhythm of temperature and displayed a mean body temperature 2°C lower than controls [24], with potential profound long-term consequences for the newborn.

Building on our previous findings [17, 21–23], here we explored the developmental impact of gestational chronodisruption on the primate adrenal function, at the molecular, morphological, and endocrine levels from early birth to young animals.

Evidence suggesting that the onset of adult diseases, such as hypertension and metabolic disorders, may originate from alterations of the hypothalamus-pituitary-adrenal (HPA) axis setting* in utero *[25–27] in conjunction with our findings led us to examine the hypothesis that gestational chronodisruption (such as exposure to constant light) has long lasting effects on adrenal function in primate newborns and infants. We tested this hypothesis in the capuchin monkey, by investigating the effects of continuous maternal exposure to constant light during the last third of gestation on cortisol and DHAS production at birth, and at one and ten months of age. We measured (i)* in vivo* plasma DHAS and cortisol levels immediately after birth and (ii) plasma DHAS and cortisol response to exogenous ACTH at one and ten months of age in infants pretreated with dexamethasone to suppress endogenous ACTH. To assess adrenal function at 10 months of age, we measured adrenal weight, mRNA, and protein expression levels of key factors in steroid synthesis (StAR and 3*β*-HSD) as well as DHAS and cortisol responses to ACTH and the circadian transducer melatonin* in vitro*

## 2. Material and Methods

### 2.1. Animals

We studied capuchin monkey (*Cebus apella*) offspring along 3 developmental stages: at birth (n=8), 1-month-old infants (n=8), and 10-month-old infants (n=8) at the Chilean Primate Center, Pontificia Universidad Católica de Chile, Santiago, Chile. For each comparison, 4 newborns were delivered by mothers which had been exposed to constant light (2,000 lux at the head level; LL group) during the last third of gestation (from about 100 days of gestation up to delivery; 57.4 ± 0.95 days), whilst the other 4 were control newborns; i.e., whose mothers had undergone pregnancy under normal 14:10 light:dark conditions of the colony (lights on at 0700 h). In the facility, females and weaned newborns were maintained in individual cages, in a room with controlled temperature and humidity; with food administered twice a day and water available* ad libitum *[23].

Maternal weight, fetal growth, and heart rate (ultrasound) were measured at 10-day intervals under i.m. Ketamine (10 mg/kg of body weight; Ketaset, Laboratorios Wyeth Inc., Santiago, Chile) as reported [23]. After delivery, LL mothers and their newborns returned to the normal photoperiod of the colony (14:10). Newborn health was controlled weekly during the first month of life and at monthly intervals thereafter by one of our team members (Natalia Méndez, DVM) and also by the colony DVM. At birth, newborns were of similar size (biparietal diameter, frontooccipital and chest circumference, femur and crown to heel length; data not shown). The rate of increase in body weight between 1 and 10 months of age was similar between the groups (Table 1).

Animal handling and care were in compliance with the recommendations of the NIH Guide for Animal Experimentation Care. The study protocol was approved and supervised by the Commission on Bioethics and Biosafety of the Facultad de Ciencias Biológicas, Pontificia Universidad Católica de Chile, Santiago, Chile (CAB-Fondecyt-1050839). The research adhered to the American Society of Primatologists (ASP) Principles for the Ethical Treatment of Nonhuman Primates (https://www.asp.org/society/resolutions/).

### 2.2. *In Vivo* Experiments

Stringent conditions were used in the design and performance of the protocols to minimize discomfort and health effects to the newborns. Venous blood samples were drawn by venipuncture using sterile 30G needles, under local (lidocaine gel) or general anesthesia (halothane). Single 0.2 ml blood samples were taken between 0900 and 1100 h at birth (3-6 hours after delivery) and at 2000 h at one and 10 months of age.* In vivo* ACTH tests were performed at 1 and 10 months of age; total volume of blood drawn was 1.2 ml. Hematocrit was measured before and after the experiments and newborn health was assessed clinically. The clock times, at which blood samples were taken in the current study, were selected according to the clock time of acrophase (clock time 0800 h) and nadir (clock time 2000 h) of cortisol circadian rhythm reported previously by us in capuchin monkey [28].

#### 2.2.1. Single Blood Samples in the Newborns at Birth, 1 Month, and 10 Months of Age

The day of birth, the mother was sedated with ketamine (10 mg/kg of body weight) and mother and newborn were removed from the cage. The newborn leg was rubbed with Lidocaine gel (4% Lidocaine gel, Laboratorio Chile S.A., Santiago, Chile) and 0.2 ml of blood was collected from one saphena vein, without separating the newborn from the mother. A similar procedure was used at one month of age (lactating newborns) to draw a blood sample at 2000 h, followed by an i.m. dexamethasone injection (2.5 mg/kg of weight, Oradexon, Organon Laboratories, Oss, Holland), using dexamethasone doses previously tested by us [28]. After these procedures, mother and infant were returned to the cage. In the newborn (1 month of age), an ACTH test was performed at 0800 h the next morning (see below). At 10 months of age (weaned infants), the infant was sedated with ketamine and removed from the cage to take the blood sample at 2000 h. Immediately after, the infant was injected with dexamethasone and returned to the cage. An ACTH test was performed at 0800 h the next morning.

#### 2.2.2. ACTH Test

At 0700 h, newborns and infants (1 and 10 months of age, respectively) were sedated with ketamine (i.m., 10 mg/kg) and atropine (0.04 mg/kg Lab. Sanderson S.A., Santiago, Chile), brought to the surgical theatre at the animal facility and anesthetized with halothane (0.5-1% in 100% oxygen). At 0800 h, a single dose of 1-24 ACTH (100 *μ*g/kg; Cortrosyn, Organon Laboratories, Oss, Holland) was injected into one saphena vein. Blood samples were taken from the other saphena vein 15 and zero min before the administration of ACTH and at 30, 60, and 120 minutes after ACTH. Plasma was separated and stored at -20°C until assayed. During the experiment, newborn wellbeing was assessed by measuring oxygen saturation, heart rate (Pulse Oximeter N-3000, Nellcor Symphony, USA), and respiratory frequency. Dexamethasone and ACTH doses were selected based on data from a previous study in adult capuchin monkeys [20]. One week after the ACTH test, the infants were sedated with ketamine and a blood sample was drawn at 0800 h before being euthanized.

### 2.3. *Ex Vivo* Studies at 10 Months of Age

The infants were euthanized by overdose of thiopental (100 mg/kg iv; Drag Pharma Invetec, Santiago, Chile). Immediately after necropsy adrenal glands were obtained in sterile condition and cut in pieces. A piece was processed in TRIzol (Invitrogen Corp., Carlsbad, CA) for total RNA and protein isolation, whilst other piece was cryopreserved for histological studies. The remaining adrenal tissue was used fresh in explants culture as previously reported [29]. Other organs were dissected and stored at -80°C in our tissue bank.

#### 2.3.1. Adrenal Explants Culture

Explants from each adrenal pair were prepared as described previously [29]. Explants were preincubated for 6 h at 37°C, 100% humidity, 5% CO_2_, and 95% air with 2 ml DMEM-F12. After 6 h preincubation, the explants were incubated by 48 h in triplicate in basal condition (medium alone; no-ACTH), in presence of 100 nM ACTH (ACTH) and 100 nM ACTH plus 100 nM melatonin (ACTH + Mel). After 48 h, the adrenal explants were harvested and weighed and the supernatants were frozen at -20°C until DHAS and cortisol measurement. In each experiment, we checked cell viability by trypan blue exclusion after dispersion with collagenase. Cell viability was measured in one explant aliquot at the beginning of the experiment, and one explant aliquot of each treatment at the end of the 48-h incubation. Percentages of dead cells (n=5 LD and n=6 LL) were 8.1 ± 3.0% for LD and 7.9 ± 4.1% for LL at the beginning of the incubation. After 48-h incubation, the percentages of dead cells were 5.7 ± 2.3 for LD and 6.0 ± 1.9% for LL.

#### 2.3.2. Semiquantification of StAR, 3*β*-HSD, and Mt1 Melatonin Receptor mRNA and StAR and 3*β*-HSD Protein Expression Levels

After total RNA isolation using TRIzol reagent (Invitrogen Corp., Carlsbad, CA), the mRNA expression levels of StAR, 3*β*-HSD, and Mt1 melatonin receptor were determined by semiquantitative RT-PCR using 18S-rRNA as housekeeping gene by a methodology previously described [29, 30]. Following total RNA isolation with TRIzol reagent, the same tissue samples were further processed to extract adrenal proteins according to the manufacturer's instructions. Protein concentration was measured by spectrophotometry at 280 nm using 1 mg/ml albumin solution as standard. The protein levels of StAR and 3*β*-HSD were measured by an amplified slot-blot technique using *β*-ACTIN as housekeeping protein, as reported previously [30].

#### 2.3.3. Histological Analysis of Adrenal Gland

Adrenal gland frozen sections were cut using a cryostat (MICROM, HM 500 OM, Walldorf, Germany) and stored at -20°C. The sections were stained with hematoxylin-eosin after drying at room temperature for 30 min, rinsing in phosphate-buffered saline and fixing for 2 min in 4% paraformaldehyde. Transmitted-light images were obtained using an Olympus microscope (Zeiss, Oberkochen, Germany).

#### 2.3.4. Hormone Assays

Cortisol and DHAS were measured in plasma and culture supernatants using RIA assays previously validated for capuchin monkey [22].

### 2.4. Data Analysis

Data are expressed as mean ± SEM. The effects of maternal exposure to constant light during late pregnancy (LL group) on plasma DHAS and cortisol concentrations in the newborn at birth and infants at 1 and 10 months of age, as well as on the expression levels of StAR, 3*β*-HSD and Mt1 mRNA and StAR and 3*β*-HSD protein in adrenal at 10 months of age were assessed by Student's t-test. The effects of ACTH on DHAS and cortisol concentration* in vivo* and* in vitro* within groups were assessed by ANOVA for repeated measures followed by the post hoc Newman-Keuls test. The effect of LL treatment on the response of DHAS and cortisol to ACTH* in vivo* was assessed by two-way ANOVA followed by the post hoc Bonferroni test. Statistical analyses were performed using GraphPad Prism software (version 7.0, GraphPad Software Inc., San Diego, CA). Results were considered significant when P values were < 0.05.

## 3. Results

Chronic maternal exposure to constant light during the last third of gestation induced pronounced and divergent changes in plasma DHAS and cortisol at birth, which tended to normalize by 10 months of age. At the adrenal level, we found a decreased steroidogenic capacity partially compensated by an increase in adrenal weight.

As reported previously [23] we did not detect effects of chronic exposure to constant light on maternal weight gain (LD: 357.6 ± 12.5 g versus LL: 369.5 ± 17.0g P=0.15 Student's t-test), plasma cortisol levels at clock time 0800h at 140 days of gestation (LD: 5.11 ± 1.07 *μ*g/ml versus LL: 4.98 ± 1.17 *μ*g/ml P=0.82 Student's t-test), and pregnancy length (157.2 ± 0.4 days versus LL: 157.8 ± 1.3 days P=0.35 Student's t-test).

### 3.1. Effect of Maternal Exposure to Constant Light during Late Gestation on Newborn and Infant Adrenal Function at Birth and One Month of Age

Immediately after birth, newborns from mothers exposed to constant light during the last third of gestation had a markedly reduced concentration of DHAS and almost twice the concentration of cortisol than control newborns (Table 2). At one month of age, plasma concentrations of DHAS in LL infants were also lower relative to control infants, whereas LL infants' cortisol concentrations were similar to those of control infants (Table 2; samples taken at clock time 2000 h), at day 29, before injecting dexamethasone.

**Figure 1:**
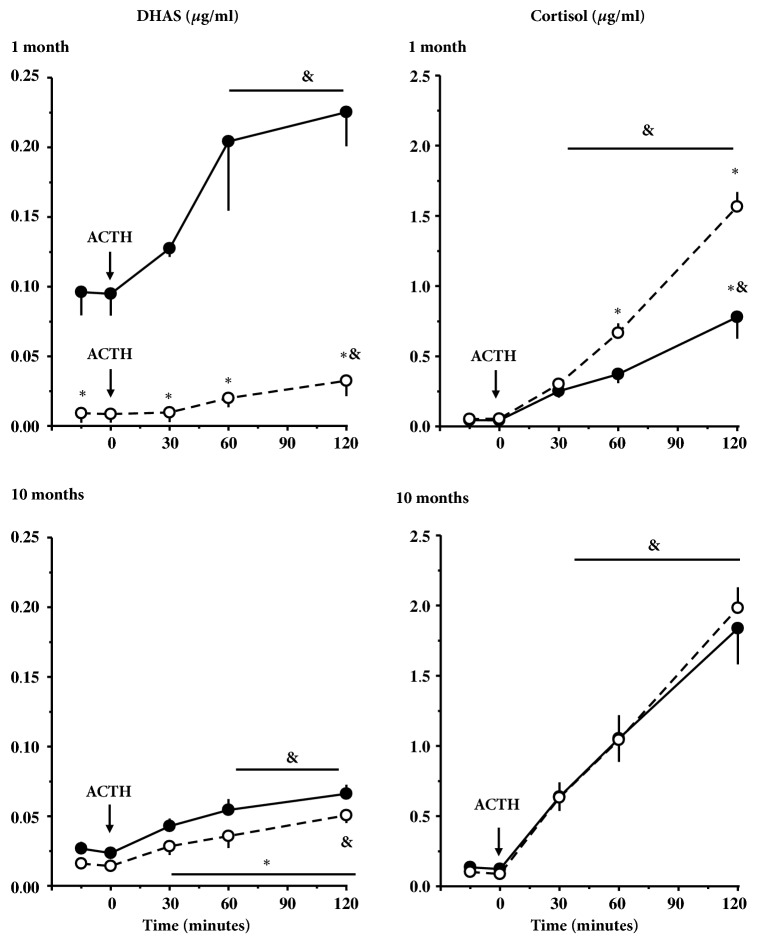
**Effect of exposure to constant light during the last third of gestation on plasma DHAS and cortisol in response to ACTH in 1-month-old and 10-month-old infants after dexamethasone treatment (see text for details).** Mean ± SEM DHAS concentration (*μ*g/mL) in (**A**) 1-month-old and (**C**) 10-month-old infants from mothers under control (n=4) and LL (n=4) conditions (14:10 light:dark, solid line, and closed circles and constant light during late gestation, broken line, and open circles, respectively). Mean ± SEM cortisol concentration (*μ*g/mL) in (**B**) 1-month-old and (**D**) 10-month-old infants from mothers under control (n=4) and LL (n=4) conditions (14:10 light:dark, solid line, and closed circles and constant light during late gestation, broken line, and open circles, respectively). *∗*Different to control (P<0.05; Student's t-test). ^&^Different to basal concentration (P< 0.05; ANOVA for repeated measures and Newman-Keuls test). Please note that for 1-month-old infants, plasma DHAS concentrations in basal conditions and in response to ACTH in LL were different to control infants (P< 0.05; two-way ANOVA for repeated measures and Bonferroni test).

As expected, dexamethasone treatment reduced DHAS and cortisol concentration the following day at clock time 0800 h to values about 1/10 lower than those at 2000 h the evening before ACTH treatment in the two groups of infants at one month of age (Table 2 and Figures 1(A) and 1(B)). ACTH induced increases of different magnitude in DHAS and cortisol in LL and control infants, with maximal responses for both steroids being observed at 90 and 120 min. The maximal DHAS concentration attained in response to ACTH was considerably smaller (about 1/10) in LL than in control infants at one month of age (Figure 1(A)); however, the percentage of increase of DHAS respect to basal values was similar in LL and control infants (304.1 ± 119.0 and 234.5 ± 25.5%, respectively). In contrast, the magnitude and the percentage increase of cortisol with respect to basal values in response to ACTH in LL infants (Figure 1(B)) were twofold higher than in control conditions (2988.1 ± 199.7 and 1751.1 ± 245.5%, P<0.05, Student's t-test).

### 3.2. Effect of Maternal Exposure to Constant Light during Late Gestation on Infant Adrenal Function at Ten Months of Age

#### 3.2.1. In Vivo Studies

Concentrations of DHAS and cortisol were measured at 0800 and 2000 h (Table 3). At 0800 h values were similar in the LL and control infants; however, there was a marked increase of DHAS at 2000 h in control infants that was absent in LL infants (Table 3). In contrast, both control and LL infants presented similar higher values of cortisol at 0800 h relative to 2000 h (Table 3).

Dexamethasone treatment effectively suppressed DHAS and cortisol to similar values in both groups of infants. ACTH induced increased DHAS and cortisol in LL and control infants, maximal responses for both steroids being observed at 120 min. As shown in (Figure 1(C)), the increase of DHAS respect to basal values was similar in the two groups (315.9 ± 34.7 and 247.2 ± 24.2%, respectively) whilst the magnitude of the response to ACTH was smaller in LL relative to control infants. In contrast, there was no difference between LL and control infants (Figure 1(D)) in percentage of increase with respect to basal values and magnitude of the cortisol response to ACTH (1585.6 ± 117.8 and 1653.5 ± 188.4%, respectively).

Comparison of the ACTH response in control animals at one and 10 months of age shows that DHAS response decreases between these ages, whereas cortisol response increases. In contrast, these developmental changes in response to ACTH were not present in LL animals.

#### 3.2.2. Ex Vivo Studies

**Figure 2:**
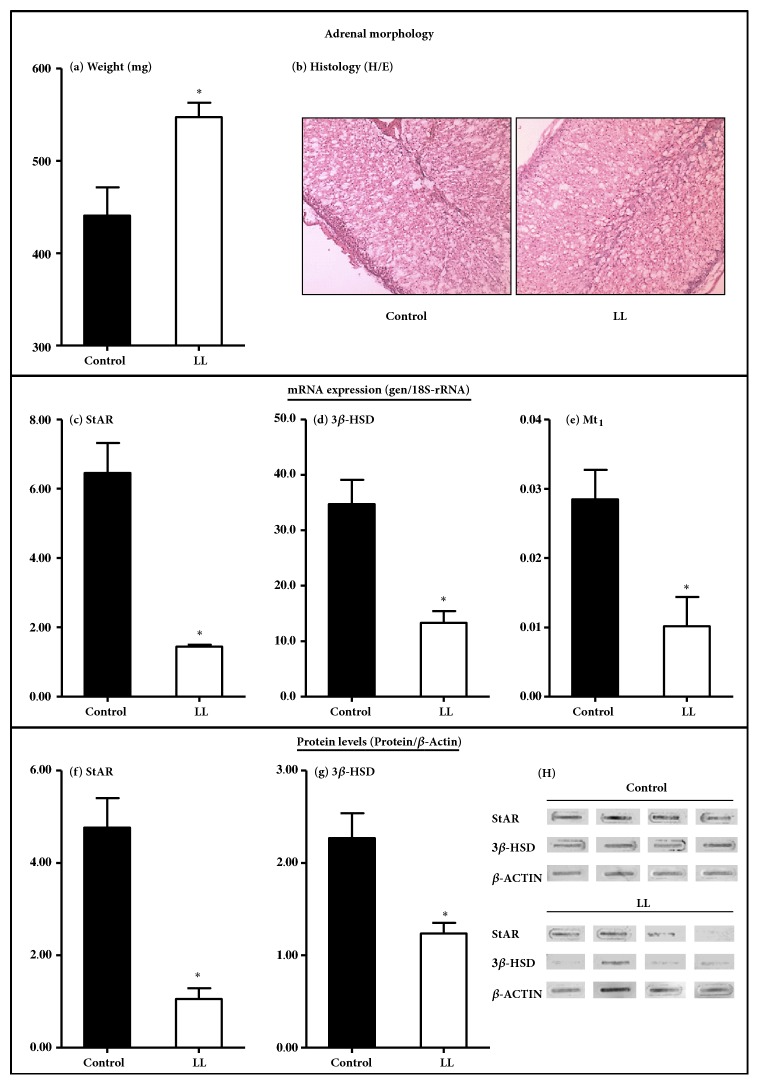
**Effects of exposure to constant light during the last third of gestation on adrenal weight (mg; mean ± SEM), histology (a and b, magnification 10X; the bar indicates 400** ***μ*** **m); adrenal expression of StAR, 3** **β** **-HSD, and Mt1 mRNA (c, d, and e, respectively), and StAR and 3** **β** **-HSD protein (f-h) in 10-month-old infants. Representative slot-blot panel (g) for StAR, 3** **β** **-HSD, and the housekeeping** **β** **-ACTIN protein**. Black bars, 10-month-old infants from mothers maintained in 14:10 light:dark cycle (control, n=4). White bars, 10-month-old infants from mothers maintained with lights continuously on from about 100 days of gestation to delivery (LL, n=4). *∗*Different to control (P<0.05; Student's t-test).

Immediately after necropsy we analyzed the effect of maternal exposure to constant light on adrenal weight and adrenal morphology. We found that adrenal weight was increased in the infants from mothers exposed to constant light, which displayed a combined adrenal weight about 30% higher than control infants (Figure 2(a)). When we analyzed the effects of treatment on overall adrenal morphology, we did not find any obvious modifications in either the adrenal zones nor in the size of the different cells (Figure 2(b)).

On the other hand, the adrenal gland from LL infants presented low levels of StAR, 3*β*-HSD, and Mt1 melatonin receptor mRNA levels (Figures 2(c)–2(e), respectively). Meanwhile, the protein levels of StAR and 3*β*-HSD were decreased (Figures 2(f)–2(h), respectively), suggesting a decreased steroidogenic capacity in the adrenals from LL infants.

#### 3.2.3. In Vitro Studies

**Figure 3:**
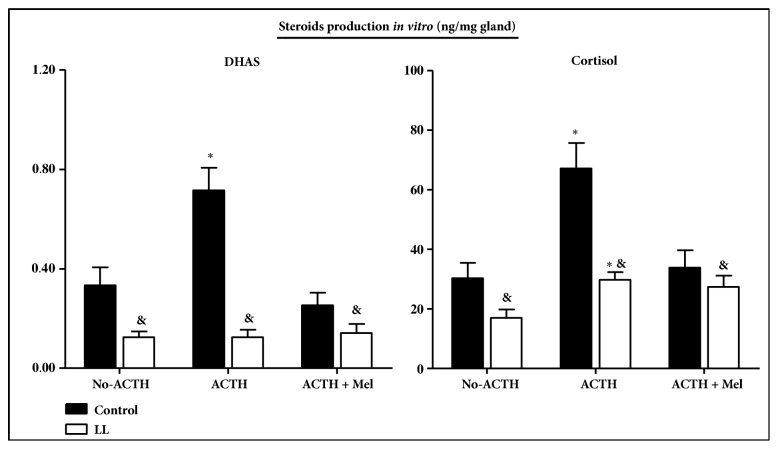
**Effect of exposure to constant light during the last third of gestation on adrenal responsiveness to ACTH* in vitro* (mean ± SEM) of DHAS (upper panel) and cortisol (lower panel) in 10-month-old infants.** Adrenal explants were incubated in medium alone (no-ACTH) or in presence of 100 nM ACTH (ACTH) during 48 h. Black bars, 10-month-old infants from mothers maintained in 14:10 light:dark cycle (control). White bars, 10-month-old infants from mothers maintained with lights continuously on from about 100 days of gestation to delivery (LL). ^&^Different to no-ACTH production (P<0.05; Student's t-test). *∗*Different to control (P< 0.05; ANOVA for repeated measures and Newman-Keuls test).

To investigate whether exposure to constant light during late gestation decreases the adrenal steroidogenic capacity in LL infants, we measured total production corrected for adrenal weight of DHAS and cortisol by adrenal explants in basal condition, in response to 100 nM ACTH and in response to 100 nM ACTH plus 100 nM melatonin (Figure 3). Basal DHAS production was much lower in adrenal explants from LL infants than in explants from control infants, whilst there was no difference in basal cortisol production. There was no increase of DHAS in response to ACTH in LL infants (Figure 3), whereas the cortisol increase in response to ACTH in LL infants was lower than in adrenal explants from control animals (Figure 3). In addition, we found that melatonin inhibited DHAS and cortisol production induced by ACTH in control conditions, an effect that was missing in the adrenal gland from LL infants (Figure 3).

## 4. Discussion

There is compelling evidence indicating that early programming or anomalous setting of the adrenal gland is key for the onset of diseases that are prevalent in our modern society. Hence, based on our previous findings in different experimental models [17, 21–23], here we explored the developmental impact of gestational chronodisruption on the primate adrenal function, at the molecular, morphological, and endocrine levels from early birth to young animals.

At birth, newborns from mothers exposed to constant light during the last third of gestation had lower DHAS and higher cortisol concentrations than control newborns, supporting early differences between these experimental groups, most probably initiated during gestation. This is consistent with our previous work indicating that LL newborns at 4-5 days of age presented higher integrated cortisol concentrations over 24 h than in age-matched control newborns [23]. Additionally, exposure to constant light had no effect on the maternal body temperature rhythm; however, a delay in the activity acrophase rhythm was noted [24]. During fetal life, the capuchin monkey presents a fetal adrenal gland with functional and morphological characteristics [23] similar to human and other primates [11, 31–33].

The differences in DHAS and cortisol levels at birth induced by maternal exposure to constant light most likely represent the decrease in fetal adrenal size due to a decrease in the fetal zone cells (DHAS producing cells) and an increase of 3*β*-HSD positive cells (cortisol producing cells), as reported previously [22].

At one month of age, when the initial effects of late gestation and delivery have been subsided, chronic exposure to constant light during the last third of gestation still had effects on adrenal function, detected on plasma DHAS levels measured at 2000 h and on the DHAS and cortisol response to ACTH* in vivo*. As observed at birth, plasma DHAS concentration remained lower than in control newborns. Nevertheless, in both groups ACTH increased DHAS in the same proportion with respect to basal values, supporting that the observed effect could be a consequence of a reduced number of cells producing DHAS. In line with this, the cortisol response to ACTH was enhanced in LL newborns. Altogether, these results are consistent with maintenance of the early maturation of the adrenal gland at one month of age, secondary to maternal exposure to constant light during gestation. The physiological role of increased plasma DHAS concentration present soon after birth is not clear. The possibility that DHAS may participate in adequate fetal adaptation for the transition from maternal to external environment must be considered. For instance, it has been demonstrated that DHAS is involved in preadipocyte maturation [34], in the response of the immune system at different levels and it can also act as antiglucocorticoid by an unknown mechanism [35]. In humans, there is compelling evidence supporting that low plasma levels of DHAS increases the incidence of obesity and cardiovascular disease [36], supporting that plasma levels of DHAS are necessary for adequate adaptation after birth. Fetal glucocorticoids (cortisol or in some species corticosterone) are important for fetal homeostasis and fetal organ maturation required for successful transition from fetus to neonate [37]. However, the impact of exposure to high glucocorticoid levels in early life remains an active field of research and debate, involving diverse effects. For instance, several studies have demonstrated that maternal or fetal glucocorticoid treatment induces hypertension in the offspring [38, 39].

At 10 months of age, seemingly the scenario was different to that found at birth and one month of age. Plasma levels of cortisol and DHAS were similar in LL and control young animal at 0800 h and the ACTH responses of these steroids were also similar, suggesting that there is an “improvement” in adrenal function* in vivo*. However, at 2000 h plasma DHAS concentration in LL young animal was much lower than in control infants whilst cortisol levels were similar. The absence of clock time differences in DHAS but not cortisol production suggests a selective and long lasting effect of maternal exposure to constant light during gestation on DHAS production and/or on the clock contained in the capuchin monkey adrenal gland. Although cortisol and DHAS circadian production is controlled by several mechanisms, it is strongly regulated by the photoperiod in adult nonhuman primates [8, 30, 40], pointing to a direct involvement of the primate adrenal clock. Our previous results in the primate fetus demonstrated the presence of a circadian rhythm of DHAS, with an acrophase opposite to the one reported in juvenile and adult humans [41] and in rhesus monkeys [40], but with similar clock time differences than those found in 10-month-old control infants reported here, in which the maximal levels of DHAS were found at 2000 h. The differences between infant capuchin and those reported in adult rhesus may reflect developmental changes in DHAS circadian rhythm, as reported in humans and sheep for cortisol rhythm or in rats for corticosterone rhythm (cited by [42]). Therefore, an effect of the maternal chronodisruption over the molecular mechanism controlling the adrenal circadian clock must be considered. Moreover, we and others reported that the integrity of the clockwork machinery in the adrenal gland is essential for an appropriate response of cortisol to ACTH in primates and rodents [8, 30, 43]. Accordingly, the results reported here could be the consequence of an alteration of the circadian clock machinery, a possibility that needs to be specifically addressed through additional experiments.

There is scant information about the role of either the maternal photoperiod or the circadian system on the long-term newborn physiology. In this context, the interesting possibility that the alteration of the adrenal steroidogenic capacity may be secondary to the lack of maternal melatonin (induced by exposure to constant light) must be considered. In fact, we previously demonstrated that melatonin shifts the circadian expression of clock genes in the fetal and adult adrenal gland under culture conditions [44–46], and maternal melatonin supplementation to mothers exposed to constant light restored the temperature rhythms in the capuchin monkey newborn [24]. Indeed, our previous work showed that all the effects found in the capuchin monkey fetal adrenal function and morphology, secondary to maternal melatonin suppression through exposure to constant light during the last third of gestation, were reversed when the mother received melatonin [46]

Next, to assess whether the differences between plasma cortisol and DHAS discussed above reflect a selective compensation for cortisol at the adrenal level, we measured adrenal weight, expression of adrenal StAR and 3*β*-HSD mRNA, and protein levels, as well as the DHAS and cortisol responses to ACTH* in vitro*. Consistent with a compensatory response, we found that adrenal weight was higher in the LL infants than in control infants and that basal cortisol production per gland was similar to that of control infants even with a lower expression of StAR and 3*β*-HSD and a decreased cortisol production per mg of tissue. However, the increase in adrenal weight did not compensate the low basal DHAS production evoked by maternal exposure to constant light during gestation. These* in vitro* studies also indicated a decreased steroidogenic capacity to respond to ACTH. These results are in contrast with the DHAS and cortisol response to ACTH* in vivo*.

The observed normal plasma levels of DHAS and cortisol in response to ACTH may imply several compensatory mechanisms operating at both intra- and extra-adrenal level. A first possibility may be a low conversion of cortisol to cortisone; however, we did not find differences between the groups, whilst the plasma concentration of cortisone and the response to ACTH were similar between the groups (data not shown). Another aspect potentially modified by maternal exposure to constant light could be the level of catecholamines. In the rhesus monkey, it has been shown that exposure to constant light induced a decrease in maternal catecholamines [47]. We did not explore the long-term effects of maternal exposure to constant light over the offspring's adrenal medulla nor did this treatment induce differences in offspring's plasma catecholamine levels in our hands (data not shown).

The present results clearly suggest that a compensatory mechanism may reside in the adrenal gland through the regulation of the enzymes involved in the synthesis of cortisol. However, factors such as altered clearance of steroid hormones or adaptive changes in innervation may also contribute to the observed effects. On the other hand, the present report demonstrates that the alteration of the photoperiod experienced by the mother during gestation induced several alterations in the adrenal function evidenced in the newborn. Recent data obtained in rats is in line with this finding, in which alternated photoperiod (shift photoperiods) during gestation induced a widespread of negative consequences in the adult offspring's health, including heart hypertrophy, decreased glucose tolerance, decreased spatial memory, and increased blood pressure and heart rate variability [17–21], opening the possibility that altered maternal photoperiod impairs normal fetal development and may induce long-term effects in the adult primate as has been reported in rodents.

In conclusion, our results support that maternal chronodisruption during late gestation has profound effects on primate adrenal gland maturation and it may set the DHAS and cortisol response to ACTH observed in capuchin infants. The long-term impact of these changes during postnatal and particularly adult life remains to be investigated.

## Figures and Tables

**Table 1:** Offspring's body weight (g; mean ± SEM) at birth and at 1 month and 10 months of age.

**Age/Groups**	**Control**	**LL**
At birth	222.5 ± 10.1	197.5 ± 15.5
1 Month	397.5 ± 5.2	370.0 ± 11.5
10 Months	992.5 ± 18.0*∗*	980.0 ± 62.7*∗*

Control: newborns and infants from mothers maintained in 14:10 light:dark cycle (n=4). LL: newborns and infants from mothers maintained with lights continuously on from about 100 days of gestation to delivery (n=4). *∗*Different to control 1-month-old infant (P<0.05 Student's t-test).

**Table 2:** Effects of exposure to constant light during late gestation on plasma DHAS and cortisol concentrations (*μ*g/mL; mean ± SEM) immediately after birth and at one month of age.

	**DHAS**		**Cortisol**	
**Age/Groups**	**Control**	**LL**	**Control**	**LL**
**Birth**	1.74 ± 0.46	0.38 ± 0.17*∗*	2.31 ± 0.29	4.34 ± 0.45*∗*
**1 month**	0.25 ± 0.01^**&**^	0.01 ± 0.03^*∗*&^	0.30 ± 0.04^**&**^	0.28 ± 0.09^**&**^

Control, newborns, and infants from mothers maintained in 14:10 light:dark cycle (n=4).

LL, newborns, and infants from mothers maintained with lights continuously on from about 100 days of gestation to delivery (n=4).

*∗*Different to control newborn/infant (P<0.05 Student's t-test).

^&^Different to plasma levels at birth (P<0.05 Student's t-test).

**Table 3:** Effects of exposure to constant light during late gestation on plasma DHAS and cortisol concentrations (mean ± SEM) at ten months of age at two clock time.

	**DHAS**		**Cortisol**	
**Clock time (h)**	**Control**	**LL**	**Control**	**LL**
**0800**	0.03 ± 0.01	0.03 ± 0.01	1.19 ± 0.25	1.64 ± 0.34
**2000**	0.20 ± 0.03*∗*	0.02 ± 0.04*∗∗*	0.55 ± 0.04*∗*	0.44 ± 0.01*∗*

Control, newborns, and infants from mothers maintained in 14:10 light:dark cycle (n=4).

LL, newborns, and infants from mothers maintained with lights continuously on from about 100 days of gestation to delivery (n=4).

*∗*Different to 0800 h (P<0.05 Student's t-test).

*∗∗*Different to control animal at 2000 h (P<0.05 Student's t-test).

## Data Availability

The results of RIA, slot-blot, and histology data used to support the findings of this study are available from the corresponding author upon request.
